# Parenting and Child/Adolescent Development: Current Updates and Global Perspectives: Second Edition

**DOI:** 10.3390/children13091213

**Published:** 2026-09-08

**Authors:** Elitsa Dimitrova, Apolinaras Zaborskis

**Affiliations:** 1 Institute for Population and Human Studies, Bulgarian Academy of Sciences & Plovdiv University Paisii Hilendarski, 1000 Sofia, Bulgaria; 2 Department of Preventive Medicine & Health Research Institute, Faculty of Public Health, Medical Academy, Lithuanian University of Health Sciences, A. Mickevičiaus 9, LT-44307 Kaunas, Lithuania; apolinaras.zaborskis@lsmu.lt

The evidence consistently indicates that parenting and family environments are important determinants of child and adolescent health. Parenting styles and emotional-regulation strategies were associated with developmental and psychological outcomes, while family support and parent–child communication were related to adolescent health-risk behaviours. This editorial synthesizes findings from eight recent studies published in Children in 2025–2026, Special Issue “Parenting and Child/Adolescent Development: Current Updates and Global Perspectives” (Second Edition), focusing on parenting practices, family relationships, parental health literacy, socioeconomic circumstances, and early-life adversity in relation to child and adolescent health and development.

## 1. Introduction

Children’s and adolescents’ health and development are shaped by interconnected biological, familial, social, socioeconomic, and environmental factors, including health and nutrition, safety and security, responsive caregiving, and opportunities for early learning [1,2,3]. These factors work together throughout childhood, influencing children’s physical growth, cognitive abilities, emotional well-being, social skills, and overall capacity to learn and thrive. Supportive relationships with parents and caregivers, access to quality healthcare and nutritious food, safe and stable living conditions, and stimulating learning environments can promote healthy development. During adolescence, relationships with peers and the wider school environment become increasingly important, with supportive family and peer relationships and positive school experiences associated with better well-being and healthier developmental outcomes [4].

Contemporary research increasingly conceptualizes child and adolescent health and development within an ecological framework, recognizing that outcomes are shaped not only by individual biological and behavioural factors but also by parenting, family relationships, socioeconomic circumstances, and the wider social environment [4,5,6].

The eight articles reviewed here, published in Children in 2025–2026, Special Issue “Parenting and Child/Adolescent Development: Current Updates and Global Perspectives” (Second Edition), illustrate this multidimensional perspective particularly well. They address parenting styles, parental perceptions, parent–child communication, family support, health-related parenting practices, parental emotion regulation strategies, digital health-information seeking, socioeconomic circumstances, and the biological embedding of early-life stress. Although the studies differ substantially in design, population, age group, and outcome measures, they converge on an important conclusion: parents and families constitute a major social context through which health risks and protective factors are transmitted to children and adolescents.

The reviewed studies can be organized into four interconnected domains: (1) parenting practices and child health and development; (2) parental perceptions, knowledge, and information-seeking; (3) family support, socioeconomic circumstances, and health behaviours; and (4) biological mechanisms linking early-life adversity with later health. Taken together, these studies provide evidence for a broad ecological model in which parental characteristics and family circumstances influence children’s and adolescents’ health both directly and indirectly through nutrition, emotional regulation, health-related behaviours, access to information, and stress-related biological pathways.

### 1.1. Parenting Practices and Child Health and Development

The study by Angeline et al. (contribution 1) provides particularly clear evidence of the connection between parenting style, physical health, and developmental outcomes. In a sample of 193 Indonesian children aged 6–59 months, 20.2% were classified as anemic. Permissive parenting was substantially more common among children with anemia and was strongly associated with anemia after adjustment for child age, maternal education, and family income (adjusted OR = 10.31). In turn, anemia was strongly associated with developmental delay (adjusted OR = 19.49). These findings are important because they place a psychosocial factor—parenting style—within a pathway traditionally dominated by biological and nutritional explanations.

The study should nevertheless be interpreted cautiously. Its cross-sectional design does not permit conclusions about causality. In particular, the association between permissive parenting and anemia may be bidirectional or confounded by unmeasured characteristics. Parents of children who have health or developmental difficulties may respond to their child’s needs with less consistent structure, potentially producing an association in which child health influences parenting rather than the reverse. Similarly, socioeconomic circumstances, dietary practices, food insecurity, maternal mental health, and access to health services may simultaneously influence parenting style and anemia.

Nevertheless, the conceptual contribution is valuable. It suggests that prevention of developmental problems may benefit from interventions that combine nutritional screening and treatment with parenting support. This integrated approach is consistent with the broader evidence reviewed by Pipelias et al. (contribution 2), who describe early-life stress and adversity as processes capable of becoming biologically embedded through neuroendocrine and epigenetic mechanisms. Their narrative review emphasizes the vulnerability of prenatal, perinatal, and early postnatal developmental periods and highlights dysregulation of the hypothalamic–pituitary–adrenal axis, altered stress responsivity, and epigenetic processes as potential mechanisms connecting early environmental exposures with lifelong health outcomes.

The juxtaposition of these two articles is particularly informative. Angeline et al. (contribution 1) demonstrate an association between a specific family characteristic and a concrete biological condition, which is then associated with developmental delay, whereas Pipelias et al. (contribution 2) provide a broader mechanistic framework explaining how adverse early environments may affect development. Together, they support a biopsychosocial interpretation of child development in which biological and psychosocial risks should not be considered separately.

### 1.2. Parental Perceptions, Health Literacy, and Information-Seeking

A second major theme concerns parents’ ability to recognize and interpret children’s health needs. Kobayashi et al. (contribution 3) examined maternal perceptions of preschool children’s body size in Japan and found substantial discrepancies between maternal perceptions and objectively assessed body size. Among children classified as having high body size, 80.8% of mothers underestimated their child’s body size, while 57.1% of mothers of children with low body size overestimated it. Use of healthcare providers as an information source was independently associated with maternal overestimation.

These findings highlight that access to health information does not necessarily guarantee accurate interpretation of children’s health indicators. Parents may possess information but lack the skills, contextual understanding, or confidence required to translate objective measurements into appropriate perceptions and decisions. Importantly, the Japanese study was conducted among preschool children aged 3–5 years, a developmental period in which routine health monitoring becomes increasingly dependent on caregivers following the last early-childhood health checkup. Thus, parental interpretation of growth information becomes especially important.

The study by Psoma et al. (contribution 4) addresses a related issue from a methodological perspective. The authors translated and culturally adapted the Children’s Health Internet Research Parental Inventory (CHIRPI) into Greek and demonstrated excellent internal consistency (Cronbach’s α = 0.91), together with moderate-to-excellent test–retest and inter-rater reliability. The instrument provides a standardized method for assessing parents’ online health-information-seeking behaviour concerning their children.

These two studies complement each other. Kobayashi et al. (contribution 3) focus on the consequences of parental interpretation, whereas Psoma et al. (contribution 4) focus on the measurement of parental information-seeking behaviour. Together they suggest that future research should move beyond simply asking whether parents obtain information. More important questions concern the quality, credibility, interpretation, and application of that information.

The growing importance of digital health information also introduces new challenges. Parents may obtain information from healthcare professionals, websites, social media, online communities, or other parents, and these sources may differ substantially in accuracy. Health literacy may moderate the extent to which information-seeking improves rather than worsens parental decision-making. The finding in the Greek study that higher education was associated with more frequent health-related internet searching and greater distress is particularly noteworthy. Education may increase access to information without necessarily protecting parents from uncertainty or anxiety.

Thus, interventions aimed at improving child health should increasingly incorporate parental health literacy and information interpretation, rather than focusing exclusively on information provision.

### 1.3. Family Support, Socioeconomic Circumstances, and Health-Related Behaviours

The articles by Bocci et al. (contribution 5), Campos et al. (contribution 6), and Dimitrova and Zaborskis (contribution 7) demonstrate the importance of the broader family environment for children’s and adolescents’ health-related behaviours. Bocci et al. (contribution 5) analysed 1427 Italian children aged 8–9 years participating in the 2023 OKkio alla SALUTE nutritional surveillance survey in Tuscany. The prevalence of overweight, including obesity, was 24%, while lower parental education and financial hardship were associated with several unhealthy behaviours. Low parental education was associated with greater consumption of sugary drinks and sedentary behaviour, whereas financial hardship was associated with inadequate fruit and vegetable consumption and greater sedentary time. Financially disadvantaged children also had higher odds of overweight.

These findings reinforce the concept that health behaviours cannot be viewed simply as individual choices. Children’s diet and physical activity are embedded in family resources, routines, parental education, and the availability and affordability of healthy alternatives. Consequently, interventions that merely provide children with information about healthy lifestyles may have limited effectiveness if the family environment constrains the ability to implement those recommendations.

The formative study by Campos et al. (contribution 6) provides a more detailed picture of these mechanisms among Hispanic families in the United States. Although the sample was very small (17 parents), the mixed-method design allowed quantitative assessment of feeding practices to be complemented by qualitative interviews. Four major themes emerged: parenting roles and routines, beliefs concerning child health and weight, beliefs regarding physical activity and screen time, and environmental and social factors affecting access to healthy food and physical activity.

The importance of this study lies less in statistical generalizability than in its contribution to intervention development. It demonstrates that culturally appropriate interventions must account for parents’ beliefs, routines, social environment, and practical constraints. The finding that parents already engaged in encouraging healthy eating and dietary variety also suggests that interventions should not simply identify deficits. Instead, they should build on existing strengths and modify specific practices that may increase obesity risk.

The study by Dimitrova and Zaborskis (contribution 7) extends the family-environment perspective into adolescence. Using 2021/2022 HBSC data from 64,349 15-year-olds, including 793 Bulgarian and 1137 Lithuanian adolescents, the authors examined associations between drunkenness, cigarette and e-cigarette smoking, cannabis use, family support, and communication with parents. Low family support showed the strongest associations with substance use, while difficulty communicating with mothers was generally more strongly associated with risk behaviours than difficulty communicating with fathers. Associations were generally stronger among girls and among Bulgarian adolescents than among Lithuanian adolescents.

This study is especially valuable because it demonstrates that the relevance of the family environment changes across developmental stages. In early childhood, parents directly control many aspects of nutrition, health care, routines, and behaviour. During adolescence, direct control diminishes, while emotional support, communication, trust, and connectedness become increasingly important. Family support may therefore operate as a protective factor even when parents no longer directly regulate adolescents’ behaviour.

The three studies together support a developmental model in which parental control and caregiving are particularly important in early childhood and adolescence, whereas relational support and communication become increasingly important during adolescence.

### 1.4. Parental Emotion Regulation and Psychological Functioning

The Polish validation study by Larionow et al. (contribution 8) adds an important psychological dimension to this literature. The Parental Assistance with Child Emotion Regulation (PACER) Questionnaire assesses ten strategies parents use to help children regulate emotions. Among 74 Polish-speaking parents, all PACER subscales showed good-to-excellent internal consistency, with Cronbach’s alpha values of at least 0.83. Adaptive strategies, such as encouraging cognitive reappraisal, were associated with more favourable parental outcomes, whereas maladaptive strategies, such as avoidance, were associated with greater parental burnout and psychopathology symptoms.

This study is methodologically different from the epidemiological studies described above, but conceptually it fits closely with them. Parenting is not only a set of observable behaviours; it is also influenced by parents’ own emotional resources and psychological functioning. Parents experiencing burnout, anxiety, depression, or poor well-being may have greater difficulty responding consistently and constructively to children’s emotional needs.

The findings therefore reinforce a central principle emerging across the eight articles: interventions directed at children may be less effective if parental functioning is ignored. Supporting children’s emotional development may require simultaneous attention to parental stress, emotion regulation, psychological well-being, and parenting skills.

The preliminary nature of the Polish study should, however, be recognized. The sample consisted of only 74 parents, and the cross-sectional design prevents causal conclusions. Larger and more diverse samples, together with longitudinal and intervention studies, are needed to establish whether improving parental emotion-regulation strategies actually reduces parental burnout and improves children’s emotional outcomes.

## 2. Methodological Considerations

Despite their common thematic focus, the reviewed studies differ considerably in methodological strength. Several use relatively large samples and validated instruments, whereas others are explicitly preliminary or formative. The Japanese study included 1358 mothers, the Italian study 1427 children, and the HBSC analysis more than 64,000 adolescents, providing relatively strong statistical power. In contrast, the Hispanic formative study included only 17 parents, and the Polish PACER study included 74 parents.

Cross-sectional designs predominate. These designs are useful for identifying associations and generating hypotheses but cannot establish temporal ordering or causality. This limitation is particularly important for studies involving parenting and child health because parent and child characteristics can influence each other. Longitudinal studies would be especially valuable for testing whether parenting practices precede changes in children’s health and behavior.

Measurement issues are also important. Several studies rely partly on self-reported parental information. Such data may be affected by recall bias, social desirability, or inaccurate perceptions. The Japanese study illustrates this problem directly: maternal perception itself was the research outcome, while objective anthropometric measurements provided an external reference. Similarly, research on parenting practices and health behaviours should ideally combine questionnaire data with objective or independently assessed measures where feasible.

A further methodological issue is the treatment of socioeconomic factors. The Italian, Indonesian, and Hispanic studies demonstrate that economic circumstances are closely connected with parenting and health behaviours. Socioeconomic status may therefore act simultaneously as a confounder, mediator, or effect modifier. Future studies should employ conceptual models that explicitly distinguish these roles rather than treating socioeconomic variables simply as adjustment covariates.

## 3. Implications

The eight studies collectively provide strong support for an ecological and developmental understanding of child and adolescent health. They demonstrate that health outcomes emerge from interactions among biological conditions, parenting practices, parental psychological functioning, health literacy, socioeconomic circumstances, family relationships, and broader social environments [7,8,9]. Several consistent implications can be drawn from the presented review in accordance with other studies.

First, parenting is a health-related exposure, not merely a background demographic characteristic. In line with other studies [10,11], this finding shows that parenting style, feeding practices, emotional regulation strategies, and communication can influence children’s health behaviours and potentially their physical and psychological development.

Second, family resources matter. Education and financial circumstances influence children’s diet, sedentary behaviour, obesity risk, and potentially their access to health-promoting opportunities. Effective prevention therefore requires structural as well as behavioural approaches [12,13].

Third, parental knowledge and information-seeking do not automatically produce appropriate health decisions. The Japanese (contribution 3) and Greek (contribution 4) studies demonstrate the need to improve not only access to information but also parents’ ability to interpret objective health indicators and evaluate online information [14,15].

Fourth, family relationships remain important throughout development, although their function changes with age. In early childhood, caregiving, supervision, and direct behavioural guidance are particularly prominent, whereas during adolescence, parent-adolescent communication, emotional support, and family connectedness remain important for health and well-being [16,17].

Finally, the narrative review (contribution 2) of early-life stress provides a biological framework linking these psychosocial exposures with longer-term health. Early adversity may become biologically embedded through dysregulation of the hypothalamic–pituitary–adrenal (HPA) axis and epigenetic mechanisms, particularly during sensitive developmental periods, thereby providing potential pathways linking early environmental exposures with longer-term health outcomes [18,19].

## 4. Conclusions

The reviewed literature suggests that contemporary child-health research should move toward integrated models that simultaneously consider parenting, family relationships, socioeconomic conditions, parental psychological functioning, health literacy, and biological vulnerability. No single determinant adequately explains children’s health trajectories. Instead, risks and protective factors accumulate and interact across developmental stages.

From a life-course/longitudinal perspective, exposures and experiences at earlier developmental stages may shape subsequent health trajectories, while the effects of later environments can modify or reinforce earlier patterns [20]. Thus, risks and protective factors may accumulate, interact, and operate differently across developmental stages and social contexts. Thus, future research would benefit from longitudinal designs, larger and more representative samples, culturally adapted measurement instruments, objective health indicators, and multilevel statistical models capable of examining mediation and moderation. Intervention research should likewise move beyond child-focused approaches toward family-centred strategies that combine parenting support, health-literacy enhancement, psychological support, nutritional and lifestyle interventions, and attention to socioeconomic barriers.

Taken together, these eight publications demonstrate a clear shift toward understanding children’s and adolescents’ health within the social and relational environments in which development occurs. Such an approach is particularly relevant for public health because it creates opportunities for prevention at multiple levels—from improving parents’ knowledge and skills to strengthening family support and addressing socioeconomic inequalities. The strongest future interventions are therefore likely to be those that recognize children and adolescents not as isolated recipients of health interventions, but as members of families and communities whose resources, relationships, behaviours, and environments jointly shape health across the life course. The papers presented in this Special Issue also suggest that interventions should be developmentally sensitive. Factors that are particularly influential during early childhood such as responsive caregiving and parental support for learning and development, remain relevant as children grow, but their relative importance may change as adolescents gain greater autonomy and become increasingly influenced by peers, schools, digital environments, and community contexts.
